# Impact of lymphovascular invasion on lymph node metastasis for patients undergoing radical prostatectomy with negative resection margin

**DOI:** 10.1186/s12885-017-3307-4

**Published:** 2017-05-08

**Authors:** Yong Jin Kang, Hyun-Soo Kim, Won Sik Jang, Jong Kyou Kwon, Cheol Yong Yoon, Joo Yong Lee, Kang Su Cho, Won Sik Ham, Young Deuk Choi

**Affiliations:** 1 Department of Urology, Urological Science Institute, Yonsei University College of Medicine, 50-1 Yonsei-ro, Seodaemun-gu, Seoul, Republic of Korea; 2 Department of Pathology, Severance Hospital, Yonsei University College of Medicine, Seoul, Republic of Korea

**Keywords:** Prostate, Radical prostatectomy, Prostate-specific antigen

## Abstract

**Background:**

The association between lymphovascular invasion and lymphatic or hematogenous metastasis has been suspected, with conflicting evidence. We have investigated the association between the risk of biochemical recurrence and lymphovascular invasion in resection margin negative patients, as well as its association with lymph node metastasis.

**Methods:**

One thousand six hundred thirty four patients who underwent radical prostatectomy from 2005 to 2014 were selected. Patients with bone or distant organ metastasis at the time of operation were excluded. Survival analysis was performed to assess biochemical recurrence, metastasis and mortality risks by Kaplan-Meier analysis and multivariate Cox proportional hazard regression. Odds of lymph node metastasis were evaluated by Logistic regression.

**Results:**

LVI was detected in 118 (7.4%) patients. The median follow-up duration was 33.1 months. In the Kaplan-Meier analysis, lymphovascular invasion was associated with significantly increased 5-year and 10-year BCR rate (60.2% vs. 39.1%, 60.2% vs. 40.1%, respectively; *p* < 0.001), 10-year bone metastasis rate and cancer specific mortality (16.9% vs. 5.1%, *p* = 0.001; 6.8% vs. 2.7%, *p* = 0.034, respectively) compared to patients without LVI. When stratified by T stage and resection margin status, lymphovascular invasion resulted in significantly increased 10-year biochemical recurrence rate in T3 patients both with and without positive surgical margin (*p* = 0.008, 0.005, respectively). In the multivariate Cox regression model lymphovascular invasion resulted in 1.4-fold BCR risk and 1.7-fold metastasis risk increase (95% CI 1.045–1.749, 1.024–2.950; *p* = 0.022, 0.040, respectively). Lymphovascular invasion was revealed to be strongly associated with lymph node metastasis in the multivariate Logistic regression (OR 4.317, 95% CI 2.092–8.910, *p* < 0.001).

**Conclusion:**

Lymphovascular invasion increases the risk of recurrence in T3 patients regardless of margin status, by accelerating lymph node metastasis and distant organ metastasis.

**Electronic supplementary material:**

The online version of this article (doi:10.1186/s12885-017-3307-4) contains supplementary material, which is available to authorized users.

## Background

The association between lymphovascular invasion and lymphatic or hematogenous metastasis has been suspected since 1994, when the College of American Pathologists recommended to routinely report lymphovascular invasion (LVI) for radical prostatectomy specimens; however, current evidence remains controversial [1–5]. There are several factors that make it difficult for LVI to establish its value as an independent prognostic factor for recurrence. Definitions of LVI vary from author to author, and there are issues of overdetection with artifacts, all of which contribute to the confusion regarding this particular pathologic finding [6]. In other malignancies, such as thyroid [7], lung [8], stomach [9], bladder [10], kidney [11], and breast cancers [12], LVI is considered as an independent prognostic factor. Regarding the prostate, despite the unclear prognostic value of LVI in prior studies conducted in the general population, more recent investigations of patients in particular stages show some promising aspects [1, 13, 14]. Although the migration of cancer cells into vessels is generally regarded as a necessary early step for nodal or distant metastasis in other cancers, evidence remains scarce for the prostate cancer [2, 15].

Under the hypothesis that LVI is associated with increased risk of recurrence by accelerating lymph node metastasis, we have analyzed the association between LVI and risk of biochemical recurrence (BCR) according to pathologic T stage and resection margin status, as well as its influence on the risk of lymph node metastasis.

## Methods

### Patient population

With approval from the institutional review board (protocol number 4–2015-0829), data were collected by reviewing medical charts in a retrospective fashion. 1634 patients who underwent the nerve-sparing radical prostatectomy by a single surgeon (Y.D.C.) at our hospital from 2005 to 2014 were selected. Informed consent from the participants was waived by the institutional review board. Patients with distant organ or bone metastasis prior to the operation (*n* = 33) or missing records (*n* = 1) were considered not appropriate for analysis leaving 1600 patients in the cohort.

### Histopathologic examination

**Fig. 1:**
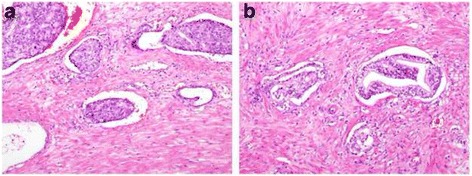
Hematoxylin-Eosin stained microscopy images (×100) of (**a**) unequivocal and (**b**) equivocal cases. Tumor emboli observed in a space with the appearance of a vessel but without a recognizable endothelial lining were considered equivocal

LVI was assessed by a single board-certified pathologist on all available hematoxylin and eosin-stained slides. LVI was defined as the invasion of vessel walls by tumor cells and/or the presence of tumor emboli within a definite endothelial-lined space, at a distance from tumors, or in the prostatic parenchyma surrounding the tumor. No attempt was made to distinguish between blood vessels and lymphatics due to the difficulty and lack of reproducibility. Only definite LVI was regarded as positive, whereas equivocal or suspected LVI (tumor emboli observed in a space with the appearance of a vessel but without a recognizable endothelial lining) was regarded as negative and considered artifacts due to peritumoral edema and tissue shrinkage (Fig. 1). Based on previous data showing that the use of immunohistochemical markers for vascular and lymphatic channels did not improve interobserver agreement in diagnosis of LVI [16], we did not perform immunohistochemical staining to determine the presence or absence of LVI. Bilateral pelvic lymph node dissection was performed at the time of radical prostatectomy by the operating surgeon’s decision. Lymph node metastasis was confirmed according to the final pathologic report.

### Postoperative follow-up

A patient was considered to have reached BCR when the postoperative prostate-specific antigen (PSA) level of 0.2 ng/mL above the nadir was detected after a nadir PSA value of 0.1 ng/mL was reached. Regional recurrence or distant organ metastasis detected on radiologic images were considered as BCR events.

### Radiologic examination

Annual or bi-annual postoperative follow-up with abdomen-pelvis computed tomography (CT) was done to detect unsuspected metastasis. Postoperatively developed lymph node enlargements were counted as a part of distant organ metastasis. Patients with BCR or relevant symptoms were screened for brain, lung, and bone metastasis with whole body positron emission CT, chest CT, and bone scintigraphy. Determination of distant organ or bone metastasis was done based on the official reading by institutional radiologists. Equivocal cases were confirmed with following positron emission tomography– CT using Fluorine-18 radiotracer or pelvis magnetic resonance image. Time to metastasis was determined as the duration from the operation date to the date of radiologic study with a first identifiable metastatic lesion.

### Statistical analysis

The Kaplan-Meier method was used to plot survival functions, and differences were assessed with the pairwise log rank test. Multivariate survival analysis for BCR was performed by constructing Cox proportional hazard regression models. Odds for lymph node metastasis were analyzed with Logistic regression. All statistical analyses were performed using Statistical Package for Social Sciences v.22.0 for Windows (SPSS, Chicago, Illinois). A *p*-value <0.05 was considered statistically significant in the current study.

## Results

### Baseline characteristics

Raw data are available as a Additional file 1. LVI was detected in 118 (7.4%) patients. Baseline characteristics of patients are as listed in Table 1. The median age was 66 (interquartile range [IQR] 61–71) and the median follow-up duration was 33.1 (IQR 18.4–53.8) months. Overall 666 (41.6%) patients were found to have BCR, and 95 (5.9%) patients developed metastasis. Forty-eight (3.0%) patients expired of prostate cancer.

**Table 1:** Baseline demographics

		Median (IQR)/n(%)
Age (years)		66(61–71)
Initial PSA (ng/mL)		8.2(5.3–15.5)
Pathologic T stage	2	741(46.3)
	3	825(51.6)
	4	33(2.1)
Lymph node metastasis	(−)	1555(97.2)
	(+)	45(2.8)
Pathologic Gleason score	≤6	434(27.1)
	7	780(48.8)
	≥8	386(24.1)
LVI	(−)	1482(92.6)
	(+)	118(7.4)
Margin status	(−)	840(52.5)
	(+)	760(47.5)
BCR	No	934(58.4)
	Yes	666(41.6)
Distant metastasis	Present	1505(94.1)
	Absent	95(5.9)
Cancer-specific mortality	Survived	1552(97.0)
	Expired	48(3.0)
Follow-up duration (months)		33.1(18.4–53.8)

*LVI* lymphovascular invasion; *PSA* prostate-specific antigen; *NHT* neoadjuvant hormone therapy; *BCR* biochemical recurrence

### Time-to-event analysis

**Fig. 2:**
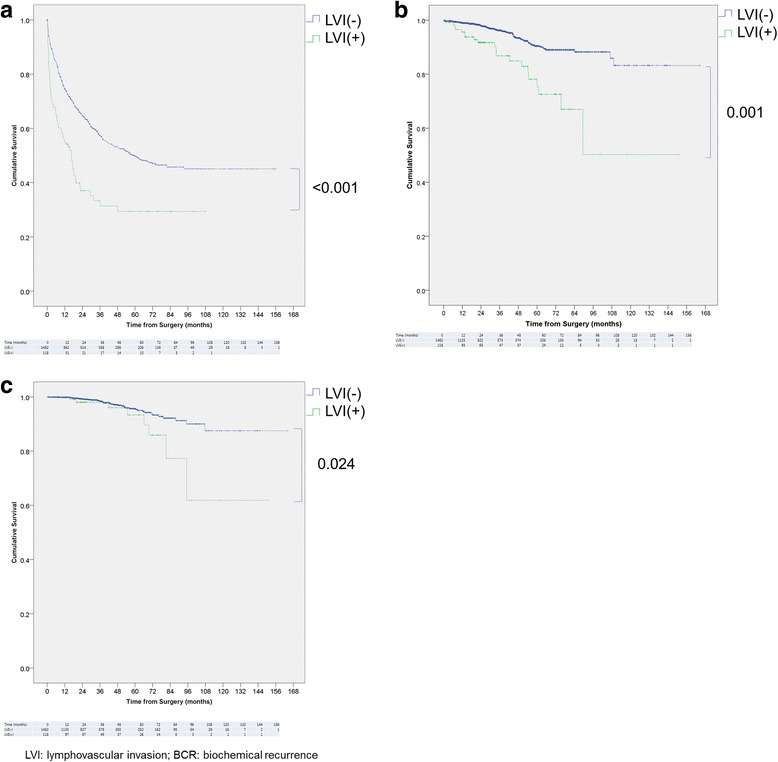
Kaplan-Meier curve for **a** BCR, **b** distant organ metastasis, **c** cancer-specific survival stratified by LVI

**Fig. 3:**
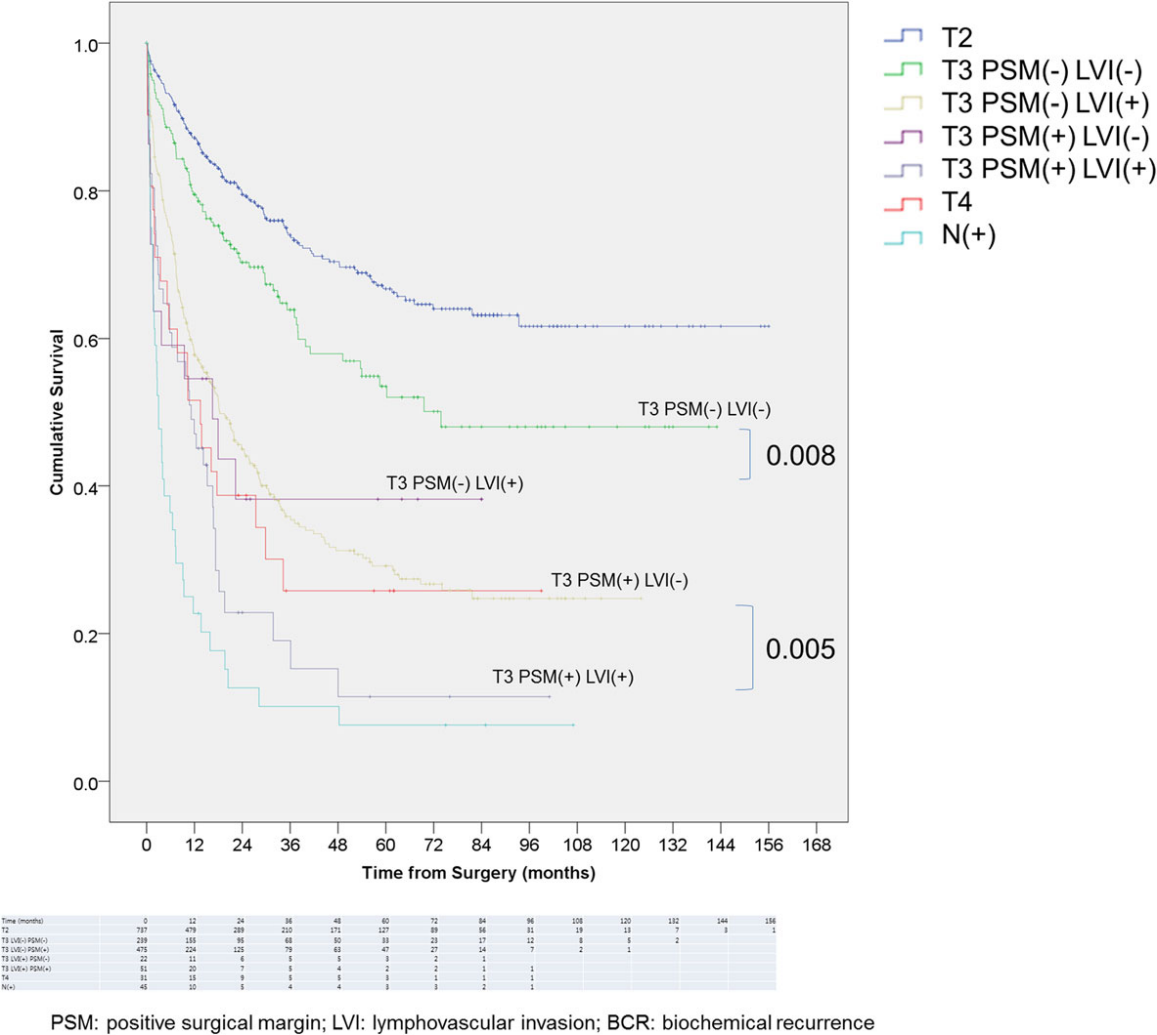
Kaplan-Meier curve for BCR stratified by T stage and PSM

Survival curves were plotted for BCR, bone metastasis, and cancer-specific survival to demonstrate the impact of LVI by Kaplan-Meier method as shown in the Fig. 2. Presence of LVI significantly increased 5-year and 10-year BCR rate compared to patients without LVI (60.2% vs. 39.1%, 60.2% vs. 40.1%, respectively; *p* < 0.001). Ten-year rate of metastasis and cancer-specific mortality was also significantly higher in LVI group (16.9% vs. 5.1%, *p* = 0.001; 6.8% vs. 2.7%, *p* = 0.034, respectively). When stratified according to T stage and surgical margin status (Fig. 3), LVI increased the risk of BCR independent of the margin status in T3 patients. Not only LVI in T3 with negative margin patients increased the 10-year BCR rate (59.1% vs. 36.4%, *p* = 0.008) to a level comparable to T3 positive margin (vs. 60.4%, *p* = 0.937) or T4 patients (vs. 71.0%, *p* = 0.658), but also in T3 with positive margin, 10-year rate of BCR for LVI patients were significantly higher than patients without LVI (76.5% vs. 59.1%, *p* = 0.005).

### Cox proportional hazard regression

As LVI is renowned for its close association with adverse prognostic factors, multivariate regression model was constructed to control for their effect. On preliminary univariate analysis, age, initial PSA ≥ 20 ng/mL, pathologic Gleason sum 7, ≥8, pathologic T stage 3, 4, PSM, lymph node metastasis and LVI showed statistically significant difference in BCR risk, as shown in Table 2. In the following multivariate analysis, LVI resulted in 1.4-fold increased risk of BCR (95% confidence interval [CI] 1.045–1.749, *p* = 0.022), when adjusted for factors found significant in the univariate analysis, such as initial PSA, pathologic T stage, pathologic Gleason sum, PSM, and lymph node metastasis. While age showed significant association in univariate analysis, it was found to be not associated with risk increase when other known prognostic factors were accounted for (*p* = 0.855). When assessed for the risk of distant organ metastasis, univariate analysis revealed age, PSA, T stage 4, Gleason score 7, ≥8, PSM, lymph node metastasis, and LVI to show association. In the multivariate model, LVI significantly increased the metastasis risk (hazard ratio [HR] 1.738, 95% CI 1.024–2.950, *p* = 0.040). There were no significant associations for age, PSA, and PSM in the model, while T stage 4, Gleason score ≥ 8, and lymph node metastasis retained statistical significance.

**Table 2:** Cox regression for BCR and distant metastasis with LVI and other parameters as covariate

		BCR				Distant metastasis			
		Univariate	Multivariate			Univariate	Multivariate		
		*p-value*	HR	95% CI	*p-value*	*p-value*	HR	95% CI	*p-value*
Age (years)		0.001*	1.001	0.990–1.012	0.855	0.048*	1.023	0.992–1.055	0.149
Initial PSA (ng/mL)	≥20	<0.001*	2.230	1.867–2.662	<0.001*	<0.001*	1.495	0.950–2.353	0.082
Pathologic T stage									
	3	<0.001*	1.522	1.253–1.849	<0.001*	0.103	0.704	0.409–1.212	0.205
	4	<0.001*	1.707	1.094–2.664	0.019*	<0.001*	2.399	1.026–5.611	0.043*
Pathologic Gleason score									
	7	<0.001*	1.953	1.521–2.508	<0.001*	<0.001*	1.123	0.566–2.229	0.741
	≥8	<0.001*	3.233	2.474–4.224	<0.001*	<0.001*	3.284	1.670–6.454	0.001*
PSM		<0.001*	2.125	1.775–2.543	<0.001*	0.005*	1.317	0.819–2.118	0.256
LVI		<0.001*	1.352	1.045–1.749	0.022*	<0.001*	1.738	1.024–2.950	0.040*
Lymph node metastasis		<0.001*	1.459	1.035–2.056	0.031*	<0.001*	5.083	2.954–8.749	<0.001*

LVI: lymphovascular invasion; PSM: positive surgical margin; PSA: prostate-specific antigen; NHT: neoadjuvant hormone therapy; HR: hazard ratio; CI: confidence interval

^*^statistically significant at *p* < 0.05

### Logistic regression for lymph node metastasis

To investigate the influence of LVI on the lymph node metastasis, logistic regression was performed (Table 3). Pathologic T stage above 3, LVI, PSM, PSA above 20 ng/mL, pathologic Gleason score above 8 were significantly associated with lymph node metastasis in the univariate analysis (all *p* < 0.001). With the parameters found significant in the univariate analysis, multivariate model was constructed. Second to the Gleason score above 8 (odds ratio [OR] 5.745, 95% CI 2.687–12.285, *p* < 0.001), LVI was associated with high odds of concurrent lymph node metastasis (OR 4.317, 95% CI 2.092–8.910, *p* < 0.001). Elevated PSA above 20 ng/mL (OR 3.208 95% CI 1.647–6.246, *p* = 0.001) and PSM (OR 3.697, 95% CI 1.462–9.351, *p* = 0.006) were also associated with lymph node metastasis. T3 stage did not show statistically significant association in the multivariate analysis (*p* = 0.165).

**Table 3:** Odds of LVI for lymph node metastasis analyzed by multivariate logistic regression

		Univariate	Multivariate		
		*p-value*	OR	95% CI	*p-value*
PSA (ng/mL)	≥20	<0.001*	3.208	1.647-6.246	0.001*
Pathologic T stage	≥3	<0.001*	2.216	0.722-6.803	0.165
Pathologic Gleason score	≥8	<0.001*	5.745	2.687-12.285	<0.001*
PSM		<0.001*	3.697	1.462-9.351	0.006*
LVI		<0.001*	4.317	2.092-8.910	<0.001*

*LVI* lymphovascular invasion; *PSM* positivie surgical margin; *PSA* prostate-specific antigen;*OR* odds ratio;*CI* confidence interval

## Discussion

Our study is, to our knowledge, the largest to demonstrate that LVI increases the recurrence risk in patients with T3 tumors independent of resection margin status. Our results indicate that increased BCR rate in LVI tumors is mainly mediated by increased lymph node metastasis, a cause different from residual cancer cells by PSM cancers. Although previous studies generally agree that LVI is associated with disease progression and aggressive tumor behavior, its value as an independent prognostic factor remains debatable. According to Loeb et al., LVI was not an independent predictor of progression in a multivariate model, although it showed a significant association with tumor volume, Gleason score > 6, PSM, extraprostatic extension (EPE), positive lymph nodes, and seminal vesicle invasion (SVI) [3]. Shariat et al., in their study involving 630 patients, have concluded that a correlation between BCR and LVI is mediated via an association with established features of biologically aggressive prostate cancer, and that LVI is a lethal phenotype of prostate cancer, leading to early metastasis and lower overall survival [2]. A close association of LVI with features of aggressive disease were highlighted again in the study by Yee et al. Whereas LVI was shown to have an independent prognostic value of its own in multivariate analysis, only marginal improvement was noted with the addition of LVI in the model, rendering the parameter not clinically meaningful [4].

Conversely, other evidence suggests that LVI is an independent prognostic factor, aside from its close association with the features of disease aggressiveness. Cheng et al. reported LVI to be an independent prognostic factor of BCR and cancer-specific survival for clinically localized prostate cancer patients, increasing BCR risk by 1.6 fold in their multivariate model (95% CI 1.12–2.38) [15]. Similarly, May et al. found that in addition to high Gleason score (HR 3.51, 95% CI 2.06–6.00), LVI is the factor that significantly increases the BCR risk for organ confined cancer patients (HR 4.39, 95% CI 2.47–7.80) [5]. Our results from Kaplan-Meier analysis indicate that LVI increases BCR rate and cancer-specific mortality up to 20% and 5%, respectively (Fig. 2). Controlling for the other known parameters, our regression model found LVI to retain statistical significance in its association with increased BCR risk (HR 1.352, 95% CI 1.045–1.749, *p* = 0.022). This disproves the belief that the increased recurrences in the LVI patients are due to the strong association with other established prognostic factors.

Especially in the cancers of T3 stage, we identified that there exists difference in the pattern of disease progression between LVI positive and negative patients. Radical prostatectomy has been not recommended on T3 patient up until recently as it was reported to be rarely curative [17]. However, from several randomized clinical trials, operation is now proven to be a valid option as it grants improved survival benefit in T3 patients [18]. Among the factors associated with recurrence risk, incomplete resection and unsuspected lymph node metastasis remains a major obstacle [17]. PSM suggests the presence of remnant cancer in the prostate bed, whether viable or not [19, 20]. It is recognized as a strong predictive factor for BCR, and attempting regional control with radiotherapy is known to yield favorable results [21]. Along with PSM, LVI is reported to increase BCR as well in T3 patients. Herman et al. evaluated pT3N0 disease and found that LVI was an independent predictor of disease recurrence [13]. Similar conclusions were drawn from the studies by Yamamoto et al. and Epstein et al., where LVI was found to be an independent prognostic factor in pT3a and pT3b tumors, respectively [1, 14]. Our results indicate that both PSM and LVI increase BCR risk, but in an independent manner. Presence of LVI significantly increased BCR rate in both positive and negative resection margin T3 groups (Fig. 3) as well as in multivariate model with PSM as a covariate (Table 2). It was shown by Mitsuzuka et al. that, LVI can increase BCR risk without PSM in T2 patients (*p* < 0.001, HR 3.809) [22]. Our result extends the finding to T3 cancers, adding to the evidence that mechanism by which cancer progresses for LVI positive cancer is different from PSM cancer where local tumor recurrence is believed to be a major cause of treatment failure [23].

LVI may serve as a portal of entry for systemic progress via regional lymph nodes. Liauw et al. [24] in the study on the role of of SVI and LVI for salvage radiotherapy patients, they demonstrated that LVI was associated with increased risk of BCR after radiotherapy (*p* = 0.019) and resulted in treatment failure in all patients, concluding that salvage radiotherapy was ineffective in LVI patients. It is likely that this is due to metastatic regions being present beyond the coverage of radiotherapy but currently, little is known about the association between LVI and metastasis regarding prostate cancer. Cheng et al. reported increased rate of LVI in lymph node metastasis patients (61% vs. 20%, *p* < 0.0001), but the evidence was inconclusive as LVI was also increased in patients with several other established prognostic factors [15]. Shariat et al. also suggested the association between LVI and lymph node metastasis by demonstrating that 5-year metastasis free rate was significantly lower as 63.0% in LVI group, compared to 96.7% in no LVI group [2]. In search of association between LVI and metastasis, we identified that LVI is associated with increased 10-year distant organ metastasis rate (16.9% vs. 5.1%, *p* = 0.001). The association was independent of other prognostic factors in the multivariate analysis (HR 1.738, 95% CI 1.024–2.950; *p* = 0.040), and we speculate that increased progression to metastatic disease stems from the early access to lymphatic channel, as LVI (OR 4.317, 95% CI 2.092–8.910, *p* < 0.001) was strongly associated with concomitant lymph node metastasis in our Logistic regression model (Table 3). Only parameter to show stronger association than LVI was Gleason score ≥ 8 (OR 5.745), which is a well-known prognostic factor of lymph node metastasis [25]. According to our results, occult metastasis should be suspected when LVI is detected, and meticulous follow-up examinations for the evidence of metastasis should be ready.

Our study has some important limitations. Firstly, no discrimination between lymphatic and vascular invasion has been made. While it is possible to identify blood vessels from the lymphatic channel by immunohistochemical analysis, its clinical significance is not clear, and we have made no effort in describing the nature of vessels [6]. In addition, due to the retrospective nature of our study, radiologic images were interpreted by several other radiologists, allowing possible inter-observer variations. Utilization of pre-set criteria in the prospective setting may improve accuracy of results.

## Conclusion

LVI increased the risk of recurrence in T3 patients independent of margin status. With its close association to lymph node metastasis, occult metastasis via lymphatic channel should be suspected in patients with LVI.

## Additional file

Additional file 1: Raw patient parameters used in the analysis. (XLSX 164 kb)

## Abbreviations

BCR: Biochemical recurrence
ECE: Extracapsular extension
LVI: Lymphovascular invasion
PSA: Prostate-specific antigen
PSM: Positive surgical margin
SVI: Seminal vesicle invasion
